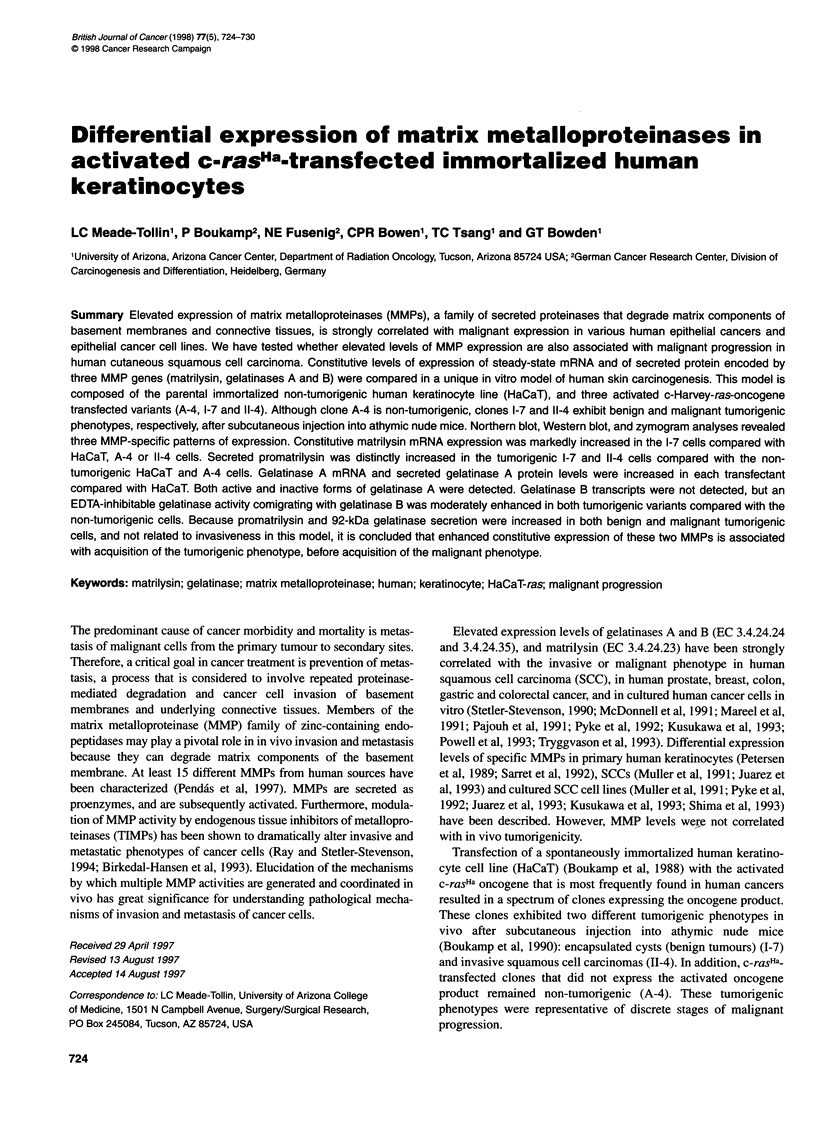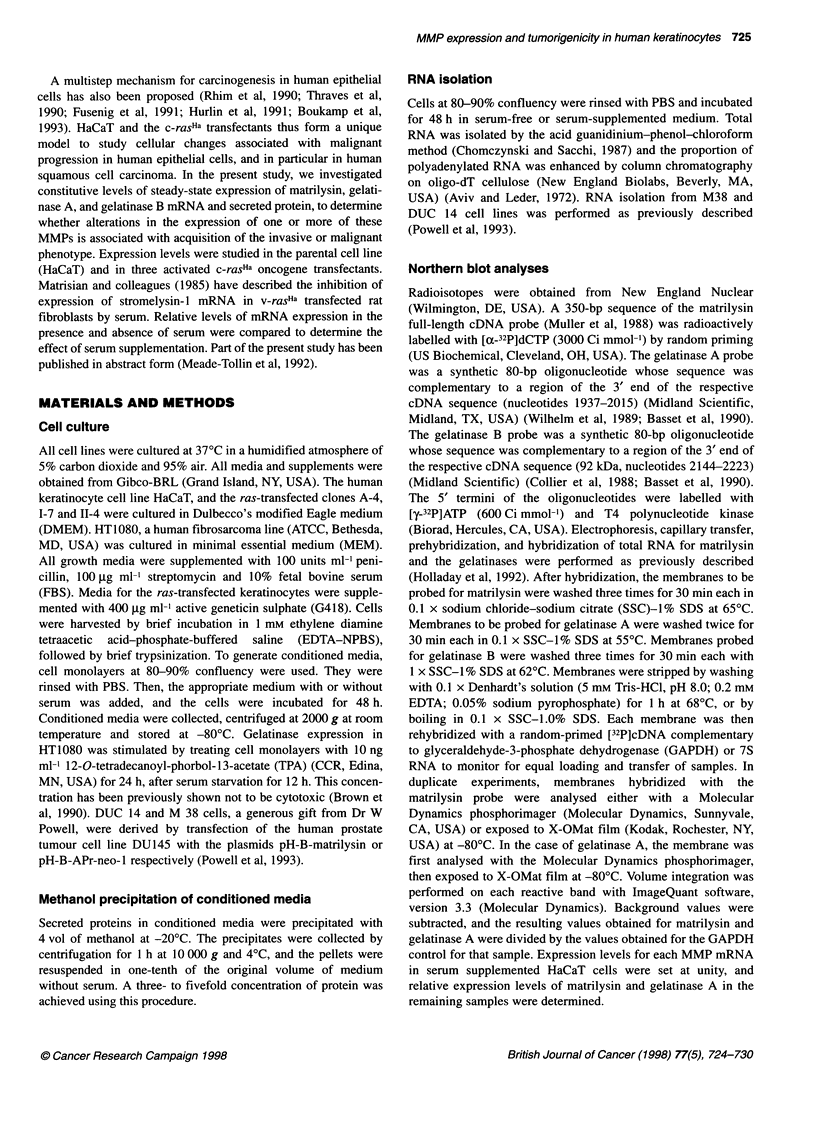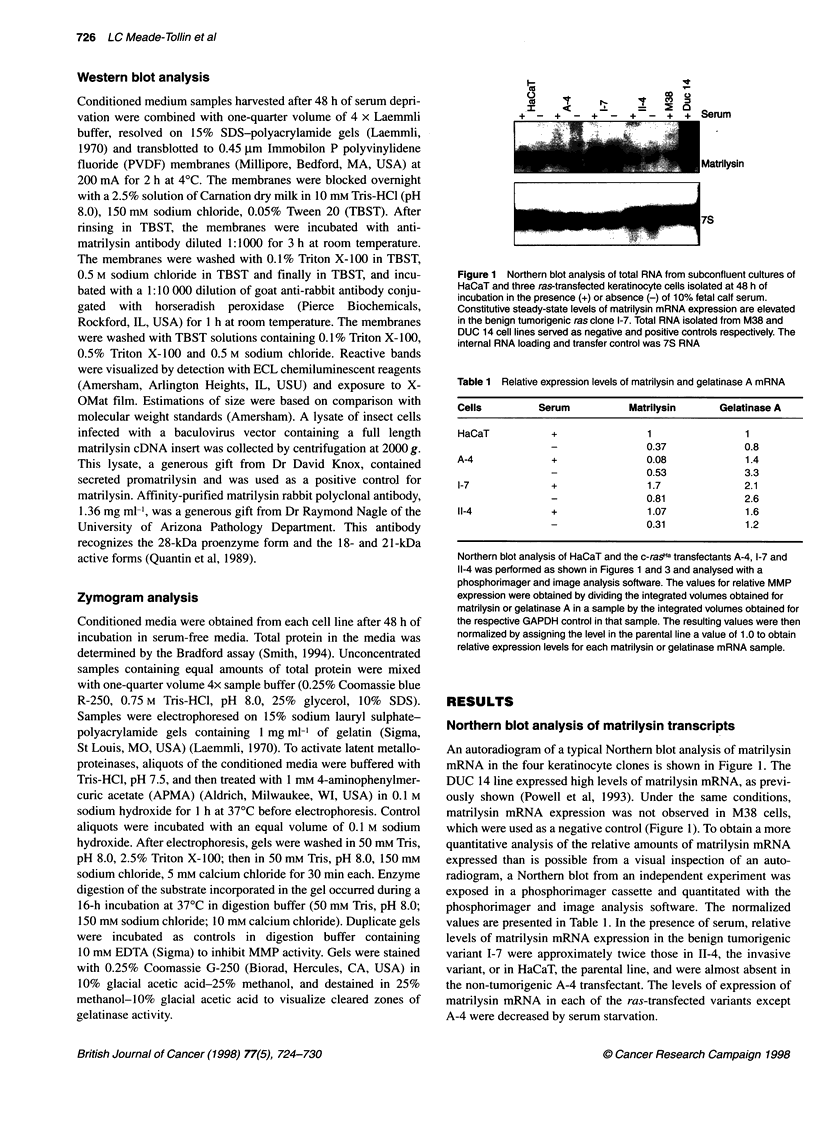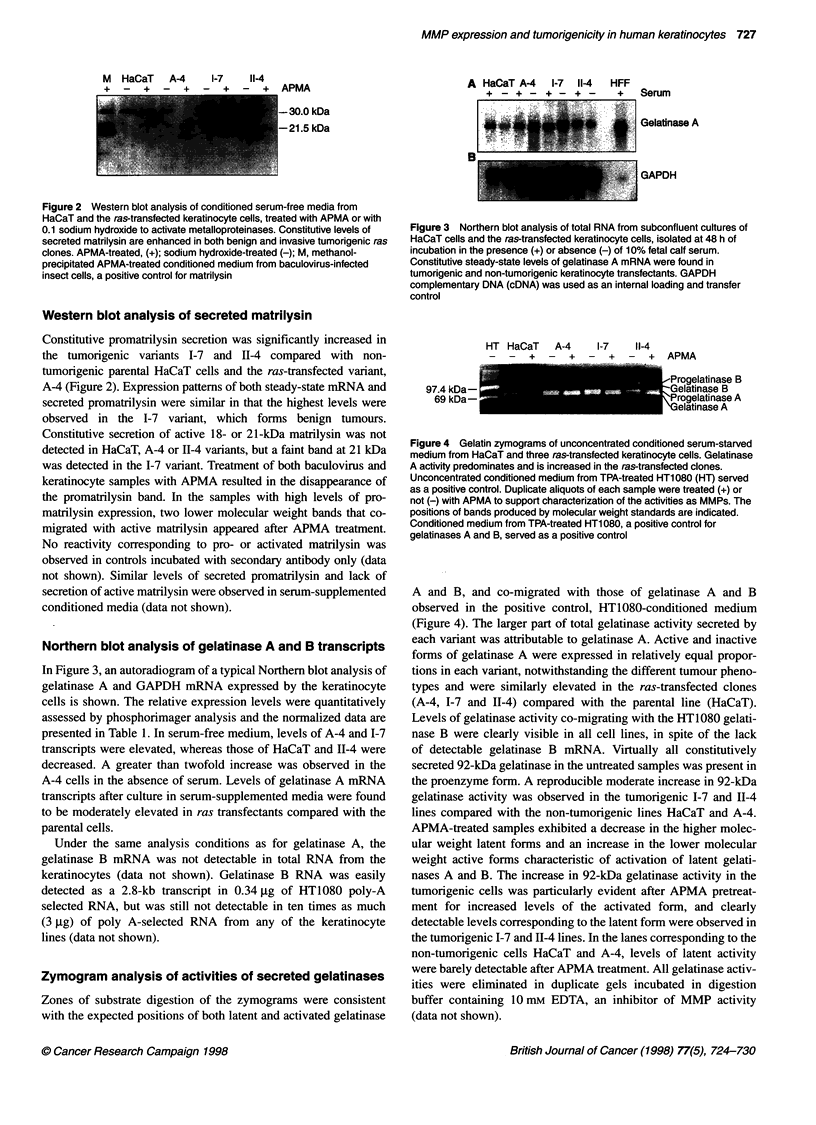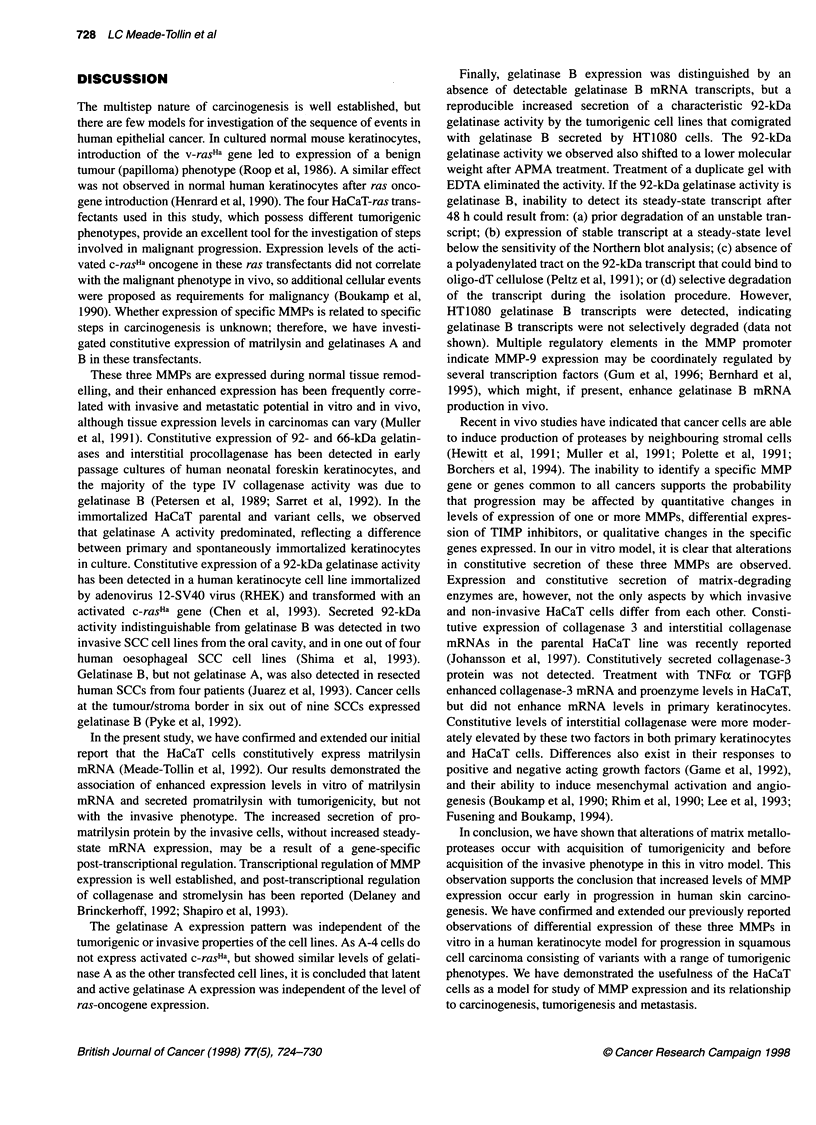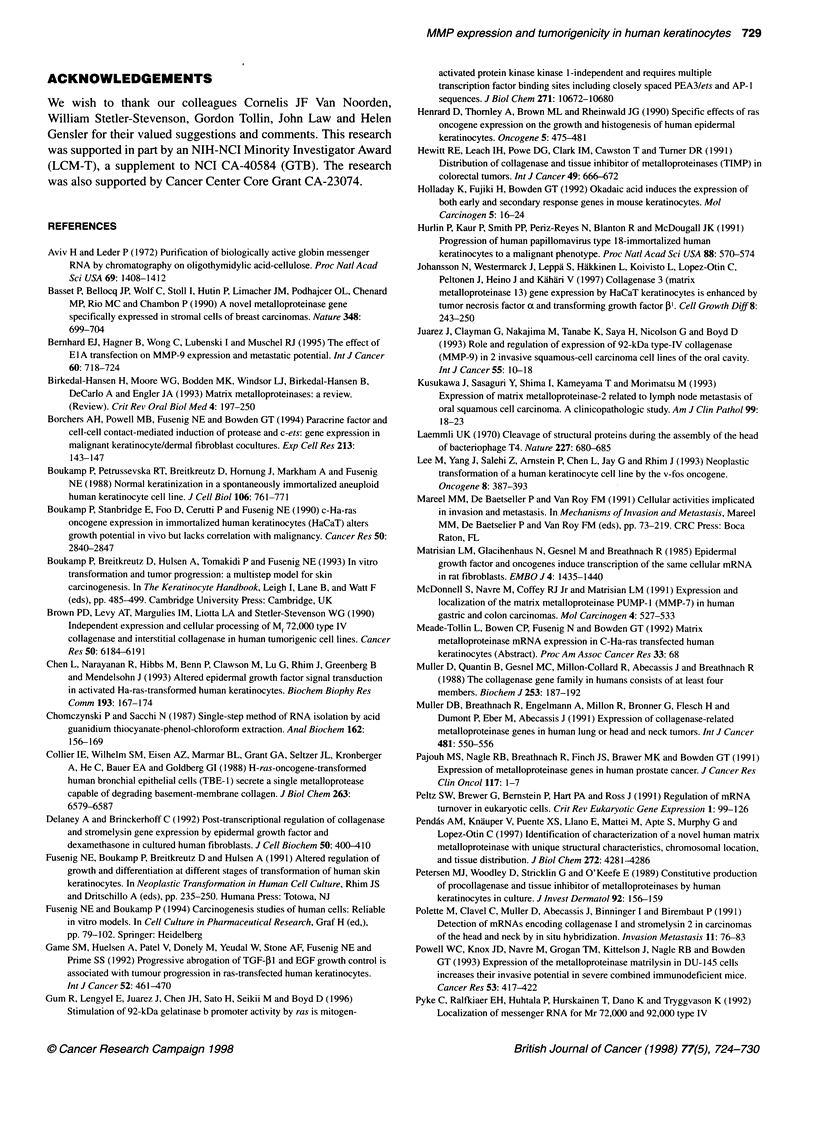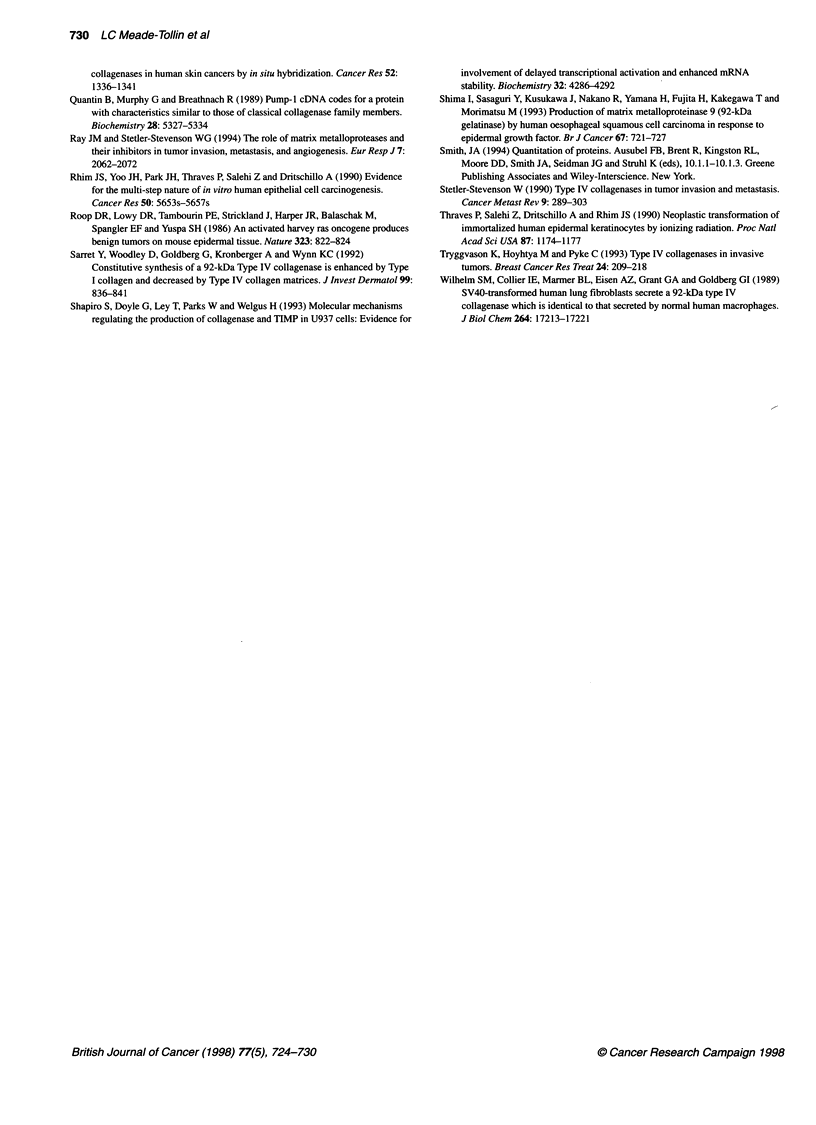# Differential expression of matrix metalloproteinases in activated c-ras-Ha-transfected immortalized human keratinocytes.

**DOI:** 10.1038/bjc.1998.119

**Published:** 1998-03

**Authors:** L. C. Meade-Tollin, P. Boukamp, N. E. Fusenig, C. P. Bowen, T. C. Tsang, G. T. Bowden

**Affiliations:** University of Arizona, Arizona Cancer Center, Department of Radiation Oncology, Tucson 85724, USA.

## Abstract

**Images:**


British Journal of Cancer (1998) 77(5), 724–730
© 1998 Cancer Research Campaign

### Differential expression of matrix metalloproteinases in activated c-*ras*$^{Ha}$-transfected immortalized human keratinocytes

**LC Meade-Tollin[1], P Boukamp[2], NE Fusenig[2], CPR Bowen[1], TC Tsang[1] and GT Bowden[1]**

[1]University of Arizona, Arizona Cancer Center, Department of Radiation Oncology, Tucson, Arizona 85724 USA; [2]German Cancer Research Center, Division of Carcinogenesis and Differentiation, Heidelberg, Germany

**Summary** Elevated expression of matrix metalloproteinases (MMPs), a family of secreted proteinases that degrade matrix components of basement membranes and connective tissues, is strongly correlated with malignant expression in various human epithelial cancers and epithelial cancer cell lines. We have tested whether elevated levels of MMP expression are also associated with malignant progression in human cutaneous squamous cell carcinoma. Constitutive levels of expression of steady-state mRNA and of secreted protein encoded by three MMP genes (matrilysin, gelatinases A and B) were compared in a unique in vitro model of human skin carcinogenesis. This model is composed of the parental immortalized non-tumorigenic human keratinocyte line (HaCaT), and three activated c-Harvey-*ras*-oncogene transfected variants (A-4, I-7 and II-4). Although clone A-4 is non-tumorigenic, clones I-7 and II-4 exhibit benign and malignant tumorigenic phenotypes, respectively, after subcutaneous injection into athymic nude mice. Northern blot, Western blot, and zymogram analyses revealed three MMP-specific patterns of expression. Constitutive matrilysin mRNA expression was markedly increased in the I-7 cells compared with HaCaT, A-4 or II-4 cells. Secreted promatrilysin was distinctly increased in the tumorigenic I-7 and II-4 cells compared with the non-tumorigenic HaCaT and A-4 cells. Gelatinase A mRNA and secreted gelatinase A protein levels were increased in each transfectant compared with HaCaT. Both active and inactive forms of gelatinase A were detected. Gelatinase B transcripts were not detected, but an EDTA-inhibitable gelatinase activity comigrating with gelatinase B was moderately enhanced in both tumorigenic variants compared with the non-tumorigenic cells. Because promatrilysin and 92-kDa gelatinase secretion were increased in both benign and malignant tumorigenic cells, and not related to invasiveness in this model, it is concluded that enhanced constitutive expression of these two MMPs is associated with acquisition of the tumorigenic phenotype, before acquisition of the malignant phenotype.

**Keywords:** matrilysin; gelatinase; matrix metalloproteinase; human; keratinocyte; HaCaT-*ras*; malignant progression

The predominant cause of cancer morbidity and mortality is metastasis of malignant cells from the primary tumour to secondary sites. Therefore, a critical goal in cancer treatment is prevention of metastasis, a process that is considered to involve repeated proteinase-mediated degradation and cancer cell invasion of basement membranes and underlying connective tissues. Members of the matrix metalloproteinase (MMP) family of zinc-containing endopeptidases may play a pivotal role in in vivo invasion and metastasis because they can degrade matrix components of the basement membrane. At least 15 different MMPs from human sources have been characterized (Pendás et al, 1997). MMPs are secreted as proenzymes, and are subsequently activated. Furthermore, modulation of MMP activity by endogenous tissue inhibitors of metalloproteinases (TIMPs) has been shown to dramatically alter invasive and metastatic phenotypes of cancer cells (Ray and Stetler-Stevenson, 1994; Birkedal-Hansen et al, 1993). Elucidation of the mechanisms by which multiple MMP activities are generated and coordinated in vivo has great significance for understanding pathological mechanisms of invasion and metastasis of cancer cells.

*Received 29 April 1997*
*Revised 13 August 1997*
*Accepted 14 August 1997*

*Correspondence to:* LC Meade-Tollin, University of Arizona College of Medicine, 1501 N Campbell Avenue, Surgery/Surgical Research, PO Box 245084, Tucson, AZ 85724, USA

Elevated expression levels of gelatinases A and B (EC 3.4.24.24 and 3.4.24.35), and matrilysin (EC 3.4.24.23) have been strongly correlated with the invasive or malignant phenotype in human squamous cell carcinoma (SCC), in human prostate, breast, colon, gastric and colorectal cancer, and in cultured human cancer cells in vitro (Stetler-Stevenson, 1990; McDonnell et al, 1991; Mareel et al, 1991; Pajouh et al, 1991; Pyke et al, 1992; Kusukawa et al, 1993; Powell et al, 1993; Tryggvason et al, 1993). Differential expression levels of specific MMPs in primary human keratinocytes (Petersen et al, 1989; Sarret et al, 1992), SCCs (Muller et al, 1991; Juarez et al, 1993) and cultured SCC cell lines (Muller et al, 1991; Pyke et al, 1992; Juarez et al, 1993; Kusukawa et al, 1993; Shima et al, 1993) have been described. However, MMP levels were not correlated with in vivo tumorigenicity.

Transfection of a spontaneously immortalized human keratinocyte cell line (HaCaT) (Boukamp et al, 1988) with the activated c-*ras*$^{Ha}$ oncogene that is most frequently found in human cancers resulted in a spectrum of clones expressing the oncogene product. These clones exhibited two different tumorigenic phenotypes in vivo after subcutaneous injection into athymic nude mice (Boukamp et al, 1990): encapsulated cysts (benign tumours) (I-7) and invasive squamous cell carcinomas (II-4). In addition, c-*ras*$^{Ha}$-transfected clones that did not express the activated oncogene product remained non-tumorigenic (A-4). These tumorigenic phenotypes were representative of discrete stages of malignant progression.

A multistep mechanism for carcinogenesis in human epithelial cells has also been proposed (Rhim et al, 1990; Thraves et al, 1990; Fusenig et al, 1991; Hurlin et al, 1991; Boukamp et al, 1993). HaCaT and the c-*ras*$^{Ha}$ transfectants thus form a unique model to study cellular changes associated with malignant progression in human epithelial cells, and in particular in human squamous cell carcinoma. In the present study, we investigated constitutive levels of steady-state expression of matrilysin, gelatinase A, and gelatinase B mRNA and secreted protein, to determine whether alterations in the expression of one or more of these MMPs is associated with acquisition of the invasive or malignant phenotype. Expression levels were studied in the parental cell line (HaCaT) and in three activated c-*ras*$^{Ha}$ oncogene transfectants. Matrisian and colleagues (1985) have described the inhibition of expression of stromelysin-1 mRNA in v-*ras*$^{Ha}$ transfected rat fibroblasts by serum. Relative levels of mRNA expression in the presence and absence of serum were compared to determine the effect of serum supplementation. Part of the present study has been published in abstract form (Meade-Tollin et al, 1992).

### MATERIALS AND METHODS

### Cell culture

All cell lines were cultured at 37°C in a humidified atmosphere of 5% carbon dioxide and 95% air. All media and supplements were obtained from Gibco-BRL (Grand Island, NY, USA). The human keratinocyte cell line HaCaT, and the *ras*-transfected clones A-4, I-7 and II-4 were cultured in Dulbecco's modified Eagle medium (DMEM). HT1080, a human fibrosarcoma line (ATCC, Bethesda, MD, USA) was cultured in minimal essential medium (MEM). All growth media were supplemented with 100 units $ml^{-1}$ penicillin, 100 $\mu g$ $ml^{-1}$ streptomycin and 10% fetal bovine serum (FBS). Media for the *ras*-transfected keratinocytes were supplemented with 400 $\mu g$ $ml^{-1}$ active geneticin sulphate (G418). Cells were harvested by brief incubation in 1 mM ethylene diamine tetraacetic acid–phosphate-buffered saline (EDTA–NPBS), followed by brief trypsinization. To generate conditioned media, cell monolayers at 80–90% confluency were used. They were rinsed with PBS. Then, the appropriate medium with or without serum was added, and the cells were incubated for 48 h. Conditioned media were collected, centrifuged at 2000 *g* at room temperature and stored at –80°C. Gelatinase expression in HT1080 was stimulated by treating cell monolayers with 10 ng $ml^{-1}$ 12-*O*-tetradecanoyl-phorbol-13-acetate (TPA) (CCR, Edina, MN, USA) for 24 h, after serum starvation for 12 h. This concentration has been previously shown not to be cytotoxic (Brown et al, 1990). DUC 14 and M 38 cells, a generous gift from Dr W Powell, were derived by transfection of the human prostate tumour cell line DU145 with the plasmids pH-B-matrilysin or pH-B-APr-neo-1 respectively (Powell et al, 1993).

### Methanol precipitation of conditioned media

Secreted proteins in conditioned media were precipitated with 4 vol of methanol at –20°C. The precipitates were collected by centrifugation for 1 h at 10 000 *g* and 4°C, and the pellets were resuspended in one-tenth of the original volume of medium without serum. A three- to fivefold concentration of protein was achieved using this procedure.

### RNA isolation

Cells at 80–90% confluency were rinsed with PBS and incubated for 48 h in serum-free or serum-supplemented medium. Total RNA was isolated by the acid guanidinium–phenol–chloroform method (Chomczynski and Sacchi, 1987) and the proportion of polyadenylated RNA was enhanced by column chromatography on oligo-dT cellulose (New England Biolabs, Beverly, MA, USA) (Aviv and Leder, 1972). RNA isolation from M38 and DUC 14 cell lines was performed as previously described (Powell et al, 1993).

### Northern blot analyses

Radioisotopes were obtained from New England Nuclear (Wilmington, DE, USA). A 350-bp sequence of the matrilysin full-length cDNA probe (Muller et al, 1988) was radioactively labelled with [$\alpha$-$^{32}$P]dCTP (3000 Ci $mmol^{-1}$) by random priming (US Biochemical, Cleveland, OH, USA). The gelatinase A probe was a synthetic 80-bp oligonucleotide whose sequence was complementary to a region of the 3′ end of the respective cDNA sequence (nucleotides 1937–2015) (Midland Scientific, Midland, TX, USA) (Wilhelm et al, 1989; Basset et al, 1990). The gelatinase B probe was a synthetic 80-bp oligonucleotide whose sequence was complementary to a region of the 3′ end of the respective cDNA sequence (92 kDa, nucleotides 2144–2223) (Midland Scientific) (Collier et al, 1988; Basset et al, 1990). The 5′ termini of the oligonucleotides were labelled with [$\gamma$-$^{32}$P]ATP (600 Ci $mmol^{-1}$) and T4 polynucleotide kinase (Biorad, Hercules, CA, USA). Electrophoresis, capillary transfer, prehybridization, and hybridization of total RNA for matrilysin and the gelatinases were performed as previously described (Holladay et al, 1992). After hybridization, the membranes to be probed for matrilysin were washed three times for 30 min each in 0.1 × sodium chloride–sodium citrate (SSC)–1% SDS at 65°C. Membranes to be probed for gelatinase A were washed twice for 30 min each in 0.1 × SSC–1% SDS at 55°C. Membranes probed for gelatinase B were washed three times for 30 min each with 1 × SSC–1% SDS at 62°C. Membranes were stripped by washing with 0.1 × Denhardt's solution (5 mM Tris-HCl, pH 8.0; 0.2 mM EDTA; 0.05% sodium pyrophosphate) for 1 h at 68°C, or by boiling in 0.1 × SSC–1.0% SDS. Each membrane was then rehybridized with a random-primed [$^{32}$P]cDNA complementary to glyceraldehyde-3-phosphate dehydrogenase (GAPDH) or 7S RNA to monitor for equal loading and transfer of samples. In duplicate experiments, membranes hybridized with the matrilysin probe were analysed either with a Molecular Dynamics phosphorimager (Molecular Dynamics, Sunnyvale, CA, USA) or exposed to X-OMat film (Kodak, Rochester, NY, USA) at –80°C. In the case of gelatinase A, the membrane was first analysed with the Molecular Dynamics phosphorimager, then exposed to X-OMat film at –80°C. Volume integration was performed on each reactive band with ImageQuant software, version 3.3 (Molecular Dynamics). Background values were subtracted, and the resulting values obtained for matrilysin and gelatinase A were divided by the values obtained for the GAPDH control for that sample. Expression levels for each MMP mRNA in serum supplemented HaCaT cells were set at unity, and relative expression levels of matrilysin and gelatinase A in the remaining samples were determined.

© Cancer Research Campaign 1998

### Western blot analysis

Conditioned medium samples harvested after 48 h of serum deprivation were combined with one-quarter volume of 4 × Laemmli buffer, resolved on 15% SDS–polyacrylamide gels (Laemmli, 1970) and transblotted to 0.45 µm Immobilon P polyvinylidene fluoride (PVDF) membranes (Millipore, Bedford, MA, USA) at 200 mA for 2 h at 4°C. The membranes were blocked overnight with a 2.5% solution of Carnation dry milk in 10 mM Tris-HCl (pH 8.0), 150 mM sodium chloride, 0.05% Tween 20 (TBST). After rinsing in TBST, the membranes were incubated with anti-matrilysin antibody diluted 1:1000 for 3 h at room temperature. The membranes were washed with 0.1% Triton X-100 in TBST, 0.5 M sodium chloride in TBST and finally in TBST, and incubated with a 1:10 000 dilution of goat anti-rabbit antibody conjugated with horseradish peroxidase (Pierce Biochemicals, Rockford, IL, USA) for 1 h at room temperature. The membranes were washed with TBST solutions containing 0.1% Triton X-100, 0.5% Triton X-100 and 0.5 M sodium chloride. Reactive bands were visualized by detection with ECL chemiluminescent reagents (Amersham, Arlington Heights, IL, USU) and exposure to X-OMat film. Estimations of size were based on comparison with molecular weight standards (Amersham). A lysate of insect cells infected with a baculovirus vector containing a full length matrilysin cDNA insert was collected by centrifugation at 2000 ***g***. This lysate, a generous gift from Dr David Knox, contained secreted promatrilysin and was used as a positive control for matrilysin. Affinity-purified matrilysin rabbit polyclonal antibody, 1.36 mg $ml^{-1}$, was a generous gift from Dr Raymond Nagle of the University of Arizona Pathology Department. This antibody recognizes the 28-kDa proenzyme form and the 18- and 21-kDa active forms (Quantin et al, 1989).

### Zymogram analysis

Conditioned media were obtained from each cell line after 48 h of incubation in serum-free media. Total protein in the media was determined by the Bradford assay (Smith, 1994). Unconcentrated samples containing equal amounts of total protein were mixed with one-quarter volume 4× sample buffer (0.25% Coomassie blue R-250, 0.75 M Tris-HCl, pH 8.0, 25% glycerol, 10% SDS). Samples were electrophoresed on 15% sodium lauryl sulphate–polyacrylamide gels containing 1 mg $ml^{-1}$ of gelatin (Sigma, St Louis, MO, USA) (Laemmli, 1970). To activate latent metalloproteinases, aliquots of the conditioned media were buffered with Tris-HCl, pH 7.5, and then treated with 1 mM 4-aminophenylmercuric acetate (APMA) (Aldrich, Milwaukee, WI, USA) in 0.1 M sodium hydroxide for 1 h at 37°C before electrophoresis. Control aliquots were incubated with an equal volume of 0.1 M sodium hydroxide. After electrophoresis, gels were washed in 50 mM Tris, pH 8.0, 2.5% Triton X-100; then in 50 mM Tris, pH 8.0, 150 mM sodium chloride, 5 mM calcium chloride for 30 min each. Enzyme digestion of the substrate incorporated in the gel occurred during a 16-h incubation at 37°C in digestion buffer (50 mM Tris, pH 8.0; 150 mM sodium chloride; 10 mM calcium chloride). Duplicate gels were incubated as controls in digestion buffer containing 10 mM EDTA (Sigma) to inhibit MMP activity. Gels were stained with 0.25% Coomassie G-250 (Biorad, Hercules, CA, USA) in 10% glacial acetic acid–25% methanol, and destained in 25% methanol–10% glacial acetic acid to visualize cleared zones of gelatinase activity.

**Figure 1** Northern blot analysis of total RNA from subconfluent cultures of HaCaT and three *ras*-transfected keratinocyte cells isolated at 48 h of incubation in the presence (+) or absence (–) of 10% fetal calf serum. Constitutive steady-state levels of matrilysin mRNA expression are elevated in the benign tumorigenic *ras* clone I-7. Total RNA isolated from M38 and DUC 14 cell lines served as negative and positive controls respectively. The internal RNA loading and transfer control was 7S RNA

**Table 1** Relative expression levels of matrilysin and gelatinase A mRNA

| Cells | Serum | Matrilysin | Gelatinase A |
|---|---|---|---|
| HaCaT | + | 1 | 1 |
| | – | 0.37 | 0.8 |
| A-4 | + | 0.08 | 1.4 |
| | – | 0.53 | 3.3 |
| I-7 | + | 1.7 | 2.1 |
| | – | 0.81 | 2.6 |
| II-4 | + | 1.07 | 1.6 |
| | – | 0.31 | 1.2 |

Northern blot analysis of HaCaT and the c-*ras*$^{Ha}$ transfectants A-4, I-7 and II-4 was performed as shown in Figures 1 and 3 and analysed with a phosphorimager and image analysis software. The values for relative MMP expression were obtained by dividing the integrated volumes obtained for matrilysin or gelatinase A in a sample by the integrated volumes obtained for the respective GAPDH control in that sample. The resulting values were then normalized by assigning the level in the parental line a value of 1.0 to obtain relative expression levels for each matrilysin or gelatinase mRNA sample.

### RESULTS

### Northern blot analysis of matrilysin transcripts

An autoradiogram of a typical Northern blot analysis of matrilysin mRNA in the four keratinocyte clones is shown in Figure 1. The DUC 14 line expressed high levels of matrilysin mRNA, as previously shown (Powell et al, 1993). Under the same conditions, matrilysin mRNA expression was not observed in M38 cells, which were used as a negative control (Figure 1). To obtain a more quantitative analysis of the relative amounts of matrilysin mRNA expressed than is possible from a visual inspection of an autoradiogram, a Northern blot from an independent experiment was exposed in a phosphorimager cassette and quantitated with the phosphorimager and image analysis software. The normalized values are presented in Table 1. In the presence of serum, relative levels of matrilysin mRNA expression in the benign tumorigenic variant I-7 were approximately twice those in II-4, the invasive variant, or in HaCaT, the parental line, and were almost absent in the non-tumorigenic A-4 transfectant. The levels of expression of matrilysin mRNA in each of the *ras*-transfected variants except A-4 were decreased by serum starvation.

© Cancer Research Campaign 1998

**Figure 2** Western blot analysis of conditioned serum-free media from HaCaT and the *ras*-transfected keratinocyte cells, treated with APMA or with 0.1 sodium hydroxide to activate metalloproteinases. Constitutive levels of secreted matrilysin are enhanced in both benign and invasive tumorigenic *ras* clones. APMA-treated, (+); sodium hydroxide-treated (–); M, methanol-precipitated APMA-treated conditioned medium from baculovirus-infected insect cells, a positive control for matrilysin

**Figure 3** Northern blot analysis of total RNA from subconfluent cultures of HaCaT cells and the *ras*-transfected keratinocyte cells, isolated at 48 h of incubation in the presence (+) or absence (–) of 10% fetal calf serum. Constitutive steady-state levels of gelatinase A mRNA were found in tumorigenic and non-tumorigenic keratinocyte transfectants. GAPDH complementary DNA (cDNA) was used as an internal loading and transfer control

### Western blot analysis of secreted matrilysin

Constitutive promatrilysin secretion was significantly increased in the tumorigenic variants I-7 and II-4 compared with non-tumorigenic parental HaCaT cells and the *ras*-transfected variant, A-4 (Figure 2). Expression patterns of both steady-state mRNA and secreted promatrilysin were similar in that the highest levels were observed in the I-7 variant, which forms benign tumours. Constitutive secretion of active 18- or 21-kDa matrilysin was not detected in HaCaT, A-4 or II-4 variants, but a faint band at 21 kDa was detected in the I-7 variant. Treatment of both baculovirus and keratinocyte samples with APMA resulted in the disappearance of the promatrilysin band. In the samples with high levels of promatrilysin expression, two lower molecular weight bands that co-migrated with active matrilysin appeared after APMA treatment. No reactivity corresponding to pro- or activated matrilysin was observed in controls incubated with secondary antibody only (data not shown). Similar levels of secreted promatrilysin and lack of secretion of active matrilysin were observed in serum-supplemented conditioned media (data not shown).

### Northern blot analysis of gelatinase A and B transcripts

In Figure 3, an autoradiogram of a typical Northern blot analysis of gelatinase A and GAPDH mRNA expressed by the keratinocyte cells is shown. The relative expression levels were quantitatively assessed by phosphorimager analysis and the normalized data are presented in Table 1. In serum-free medium, levels of A-4 and I-7 transcripts were elevated, whereas those of HaCaT and II-4 were decreased. A greater than twofold increase was observed in the A-4 cells in the absence of serum. Levels of gelatinase A mRNA transcripts after culture in serum-supplemented media were found to be moderately elevated in *ras* transfectants compared with the parental cells.

Under the same analysis conditions as for gelatinase A, the gelatinase B mRNA was not detectable in total RNA from the keratinocytes (data not shown). Gelatinase B RNA was easily detected as a 2.8-kb transcript in 0.34 μg of HT1080 poly-A selected RNA, but was still not detectable in ten times as much (3 μg) of poly A-selected RNA from any of the keratinocyte lines (data not shown).

### Zymogram analysis of activities of secreted gelatinases

Zones of substrate digestion of the zymograms were consistent with the expected positions of both latent and activated gelatinase A and B, and co-migrated with those of gelatinase A and B observed in the positive control, HT1080-conditioned medium (Figure 4). The larger part of total gelatinase activity secreted by each variant was attributable to gelatinase A. Active and inactive forms of gelatinase A were expressed in relatively equal proportions in each variant, notwithstanding the different tumour phenotypes and were similarly elevated in the *ras*-transfected clones (A-4, I-7 and II-4) compared with the parental line (HaCaT). Levels of gelatinase activity co-migrating with the HT1080 gelatinase B were clearly visible in all cell lines, in spite of the lack of detectable gelatinase B mRNA. Virtually all constitutively secreted 92-kDa gelatinase in the untreated samples was present in the proenzyme form. A reproducible moderate increase in 92-kDa gelatinase activity was observed in the tumorigenic I-7 and II-4 lines compared with the non-tumorigenic lines HaCaT and A-4. APMA-treated samples exhibited a decrease in the higher molecular weight latent forms and an increase in the lower molecular weight active forms characteristic of activation of latent gelatinases A and B. The increase in 92-kDa gelatinase activity in the tumorigenic cells was particularly evident after APMA pretreatment for increased levels of the activated form, and clearly detectable levels corresponding to the latent form were observed in the tumorigenic I-7 and II-4 lines. In the lanes corresponding to the non-tumorigenic cells HaCaT and A-4, levels of latent activity were barely detectable after APMA treatment. All gelatinase activities were eliminated in duplicate gels incubated in digestion buffer containing 10 mM EDTA, an inhibitor of MMP activity (data not shown).

**Figure 4** Gelatin zymograms of unconcentrated conditioned serum-starved medium from HaCaT and three *ras*-transfected keratinocyte cells. Gelatinase A activity predominates and is increased in the *ras*-transfected clones. Unconcentrated conditioned medium from TPA-treated HT1080 (HT) served as a positive control. Duplicate aliquots of each sample were treated (+) or not (–) with APMA to support characterization of the activities as MMPs. The positions of bands produced by molecular weight standards are indicated. Conditioned medium from TPA-treated HT1080, a positive control for gelatinases A and B, served as a positive control

© Cancer Research Campaign 1998

### DISCUSSION

The multistep nature of carcinogenesis is well established, but there are few models for investigation of the sequence of events in human epithelial cancer. In cultured normal mouse keratinocytes, introduction of the v-$ras^{Ha}$ gene led to expression of a benign tumour (papilloma) phenotype (Roop et al, 1986). A similar effect was not observed in normal human keratinocytes after *ras* oncogene introduction (Henrard et al, 1990). The four HaCaT-*ras* transfectants used in this study, which possess different tumorigenic phenotypes, provide an excellent tool for the investigation of steps involved in malignant progression. Expression levels of the activated c-$ras^{Ha}$ oncogene in these *ras* transfectants did not correlate with the malignant phenotype in vivo, so additional cellular events were proposed as requirements for malignancy (Boukamp et al, 1990). Whether expression of specific MMPs is related to specific steps in carcinogenesis is unknown; therefore, we have investigated constitutive expression of matrilysin and gelatinases A and B in these transfectants.

These three MMPs are expressed during normal tissue remodelling, and their enhanced expression has been frequently correlated with invasive and metastatic potential in vitro and in vivo, although tissue expression levels in carcinomas can vary (Muller et al, 1991). Constitutive expression of 92- and 66-kDa gelatinases and interstitial procollagenase has been detected in early passage cultures of human neonatal foreskin keratinocytes, and the majority of the type IV collagenase activity was due to gelatinase B (Petersen et al, 1989; Sarret et al, 1992). In the immortalized HaCaT parental and variant cells, we observed that gelatinase A activity predominated, reflecting a difference between primary and spontaneously immortalized keratinocytes in culture. Constitutive expression of a 92-kDa gelatinase activity has been detected in a human keratinocyte cell line immortalized by adenovirus 12-SV40 virus (RHEK) and transformed with an activated c-$ras^{Ha}$ gene (Chen et al, 1993). Secreted 92-kDa activity indistinguishable from gelatinase B was detected in two invasive SCC cell lines from the oral cavity, and in one out of four human oesophageal SCC cell lines (Shima et al, 1993). Gelatinase B, but not gelatinase A, was also detected in resected human SCCs from four patients (Juarez et al, 1993). Cancer cells at the tumour/stroma border in six out of nine SCCs expressed gelatinase B (Pyke et al, 1992).

In the present study, we have confirmed and extended our initial report that the HaCaT cells constitutively express matrilysin mRNA (Meade-Tollin et al, 1992). Our results demonstrated the association of enhanced expression levels in vitro of matrilysin mRNA and secreted promatrilysin with tumorigenicity, but not with the invasive phenotype. The increased secretion of promatrilysin protein by the invasive cells, without increased steady-state mRNA expression, may be a result of a gene-specific post-transcriptional regulation. Transcriptional regulation of MMP expression is well established, and post-transcriptional regulation of collagenase and stromelysin has been reported (Delaney and Brinckerhoff, 1992; Shapiro et al, 1993).

The gelatinase A expression pattern was independent of the tumorigenic or invasive properties of the cell lines. As A-4 cells do not express activated c-$ras^{Ha}$, but showed similar levels of gelatinase A as the other transfected cell lines, it is concluded that latent and active gelatinase A expression was independent of the level of *ras*-oncogene expression.

Finally, gelatinase B expression was distinguished by an absence of detectable gelatinase B mRNA transcripts, but a reproducible increased secretion of a characteristic 92-kDa gelatinase activity by the tumorigenic cell lines that comigrated with gelatinase B secreted by HT1080 cells. The 92-kDa gelatinase activity we observed also shifted to a lower molecular weight after APMA treatment. Treatment of a duplicate gel with EDTA eliminated the activity. If the 92-kDa gelatinase activity is gelatinase B, inability to detect its steady-state transcript after 48 h could result from: (a) prior degradation of an unstable transcript; (b) expression of stable transcript at a steady-state level below the sensitivity of the Northern blot analysis; (c) absence of a polyadenylated tract on the 92-kDa transcript that could bind to oligo-dT cellulose (Peltz et al, 1991); or (d) selective degradation of the transcript during the isolation procedure. However, HT1080 gelatinase B transcripts were detected, indicating gelatinase B transcripts were not selectively degraded (data not shown). Multiple regulatory elements in the MMP promoter indicate MMP-9 expression may be coordinately regulated by several transcription factors (Gum et al, 1996; Bernhard et al, 1995), which might, if present, enhance gelatinase B mRNA production in vivo.

Recent in vivo studies have indicated that cancer cells are able to induce production of proteases by neighbouring stromal cells (Hewitt et al, 1991; Muller et al, 1991; Polette et al, 1991; Borchers et al, 1994). The inability to identify a specific MMP gene or genes common to all cancers supports the probability that progression may be affected by quantitative changes in levels of expression of one or more MMPs, differential expression of TIMP inhibitors, or qualitative changes in the specific genes expressed. In our in vitro model, it is clear that alterations in constitutive secretion of these three MMPs are observed. Expression and constitutive secretion of matrix-degrading enzymes are, however, not the only aspects by which invasive and non-invasive HaCaT cells differ from each other. Constitutive expression of collagenase 3 and interstitial collagenase mRNAs in the parental HaCaT line was recently reported (Johansson et al, 1997). Constitutively secreted collagenase-3 protein was not detected. Treatment with TNF$\alpha$ or TGF$\beta$ enhanced collagenase-3 mRNA and proenzyme levels in HaCaT, but did not enhance mRNA levels in primary keratinocytes. Constitutive levels of interstitial collagenase were more moderately elevated by these two factors in both primary keratinocytes and HaCaT cells. Differences also exist in their responses to positive and negative acting growth factors (Game et al, 1992), and their ability to induce mesenchymal activation and angiogenesis (Boukamp et al, 1990; Rhim et al, 1990; Lee et al, 1993; Fusening and Boukamp, 1994).

In conclusion, we have shown that alterations of matrix metalloproteases occur with acquisition of tumorigenicity and before acquisition of the invasive phenotype in this in vitro model. This observation supports the conclusion that increased levels of MMP expression occur early in progression in human skin carcinogenesis. We have confirmed and extended our previously reported observations of differential expression of these three MMPs in vitro in a human keratinocyte model for progression in squamous cell carcinoma consisting of variants with a range of tumorigenic phenotypes. We have demonstrated the usefulness of the HaCaT cells as a model for study of MMP expression and its relationship to carcinogenesis, tumorigenesis and metastasis.

© Cancer Research Campaign 1998

### ACKNOWLEDGEMENTS

We wish to thank our colleagues Cornelis JF Van Noorden, William Stetler-Stevenson, Gordon Tollin, John Law and Helen Gensler for their valued suggestions and comments. This research was supported in part by an NIH-NCI Minority Investigator Award (LCM-T), a supplement to NCI CA-40584 (GTB). The research was also supported by Cancer Center Core Grant CA-23074.

### REFERENCES

Aviv H and Leder P (1972) Purification of biologically active globin messenger RNA by chromatography on oligothymidylic acid-cellulose. *Proc Natl Acad Sci USA* **69**: 1408–1412

Basset P, Bellocq JP, Wolf C, Stoll I, Hutin P, Limacher JM, Podhajcer OL, Chenard MP, Rio MC and Chambon P (1990) A novel metalloproteinase gene specifically expressed in stromal cells of breast carcinomas. *Nature* **348**: 699–704

Bernhard EJ, Hagner B, Wong C, Lubenski I and Muschel RJ (1995) The effect of E1A transfection on MMP-9 expression and metastatic potential. *Int J Cancer* **60**: 718–724

Birkedal-Hansen H, Moore WG, Bodden MK, Windsor LJ, Birkedal-Hansen B, DeCarlo A and Engler JA (1993) Matrix metalloproteinases: a review. (Review). *Crit Rev Oral Biol Med* **4**: 197–250

Borchers AH, Powell MB, Fusenig NE and Bowden GT (1994) Paracrine factor and cell-cell contact-mediated induction of protease and c-*ets*: gene expression in malignant keratinocyte/dermal fibroblast cocultures. *Exp Cell Res* **213**: 143–147

Boukamp P, Petrussevska RT, Breitkreutz D, Hornung J, Markham A and Fusenig NE (1988) Normal keratinization in a spontaneously immortalized aneuploid human keratinocyte cell line. *J Cell Biol* **106**: 761–771

Boukamp P, Stanbridge E, Foo D, Cerutti P and Fusenig NE (1990) c-Ha-ras oncogene expression in immortalized human keratinocytes (HaCaT) alters growth potential in vivo but lacks correlation with malignancy. *Cancer Res* **50**: 2840–2847

Boukamp P, Breitkreutz D, Hulsen A, Tomakidi P and Fusenig NE (1993) In vitro transformation and tumor progression: a multistep model for skin carcinogenesis. In *The Keratinocyte Handbook*, Leigh I, Lane B, and Watt F (eds), pp. 485–499. Cambridge University Press: Cambridge, UK

Brown PD, Levy AT, Margulies IM, Liotta LA and Stetler-Stevenson WG (1990) Independent expression and cellular processing of $M_r$ 72,000 type IV collagenase and interstitial collagenase in human tumorigenic cell lines. *Cancer Res* **50**: 6184–6191

Chen L, Narayanan R, Hibbs M, Benn P, Clawson M, Lu G, Rhim J, Greenberg B and Mendelsohn J (1993) Altered epidermal growth factor signal transduction in activated Ha-ras-transformed human keratinocytes. *Biochem Biophy Res Comm* **193**: 167–174

Chomczynski P and Sacchi N (1987) Single-step method of RNA isolation by acid guanidium thiocyanate-phenol-chloroform extraction. *Anal Biochem* **162**: 156–169

Collier IE, Wilhelm SM, Eisen AZ, Marmar BL, Grant GA, Seltzer JL, Kronberger A, He C, Bauer EA and Goldberg GI (1988) H-*ras*-oncogene-transformed human bronchial epithelial cells (TBE-1) secrete a single metalloprotease capable of degrading basement-membrane collagen. *J Biol Chem* **263**: 6579–6587

Delaney A and Brinckerhoff C (1992) Post-transcriptional regulation of collagenase and stromelysin gene expression by epidermal growth factor and dexamethasone in cultured human fibroblasts. *J Cell Biochem* **50**: 400–410

Fusenig NE, Boukamp P, Breitkreutz D and Hulsen A (1991) Altered regulation of growth and differentiation at different stages of transformation of human skin keratinocytes. In *Neoplastic Transformation in Human Cell Culture*, Rhim JS and Dritschillo A (eds), pp. 235–250. Humana Press: Totowa, NJ

Fusenig NE and Boukamp P (1994) Carcinogenesis studies of human cells: Reliable in vitro models. In *Cell Culture in Pharmaceutical Research*, Graf H (ed,), pp. 79–102. Springer: Heidelberg

Game SM, Huelsen A, Patel V, Donely M, Yeudal W, Stone AF, Fusenig NE and Prime SS (1992) Progressive abrogation of TGF-β1 and EGF growth control is associated with tumour progression in ras-transfected human keratinocytes. *Int J Cancer* **52**: 461–470

Gum R, Lengyel E, Juarez J, Chen JH, Sato H, Seikii M and Boyd D (1996) Stimulation of 92-kDa gelatinase b promoter activity by *ras* is mitogen-activated protein kinase kinase 1-independent and requires multiple transcription factor binding sites including closely spaced PEA3/*ets* and AP-1 sequences. *J Biol Chem* **271**: 10672–10680

Henrard D, Thornley A, Brown ML and Rheinwald JG (1990) Specific effects of ras oncogene expression on the growth and histogenesis of human epidermal keratinocytes. *Oncogene* **5**: 475–481

Hewitt RE, Leach IH, Powe DG, Clark IM, Cawston T and Turner DR (1991) Distribution of collagenase and tissue inhibitor of metalloproteinases (TIMP) in colorectal tumors. *Int J Cancer* **49**: 666–672

Holladay K, Fujiki H, Bowden GT (1992) Okadaic acid induces the expression of both early and secondary response genes in mouse keratinocytes. *Mol Carcinogen* **5**: 16–24

Hurlin P, Kaur P, Smith PP, Periz-Reyes N, Blanton R and McDougall JK (1991) Progression of human papillomavirus type 18-immortalized human keratinocytes to a malignant phenotype. *Proc Natl Acad Sci USA* **88**: 570–574

Johansson N, Westermarck J, Leppä S, Häkkinen L, Koivisto L, Lopez-Otin C, Peltonen J, Heino J and Kähäri V (1997) Collagenase 3 (matrix metalloproteinase 13) gene expression by HaCaT keratinocytes is enhanced by tumor necrosis factor α and transforming growth factor β[1]. *Cell Growth Diff* **8**: 243–250

Juarez J, Clayman G, Nakajima M, Tanabe K, Saya H, Nicolson G and Boyd D (1993) Role and regulation of expression of 92-kDa type-IV collagenase (MMP-9) in 2 invasive squamous-cell carcinoma cell lines of the oral cavity. *Int J Cancer* **55**: 10–18

Kusukawa J, Sasaguri Y, Shima I, Kameyama T and Morimatsu M (1993) Expression of matrix metalloproteinase-2 related to lymph node metastasis of oral squamous cell carcinoma. A clinicopathologic study. *Am J Clin Pathol* **99**: 18–23

Laemmli UK (1970) Cleavage of structural proteins during the assembly of the head of bacteriophage T4. *Nature* **227**: 680–685

Lee M, Yang J, Salehi Z, Arnstein P, Chen L, Jay G and Rhim J (1993) Neoplastic transformation of a human keratinocyte cell line by the v-fos oncogene. *Oncogene* **8**: 387–393

Mareel MM, De Baetseller P and Van Roy FM (1991) Cellular activities implicated in invasion and metastasis. In *Mechanisms of Invasion and Metastasis*, Mareel MM, De Baetselier P and Van Roy FM (eds), pp. 73–219. CRC Press: Boca Raton, FL

Matrisian LM, Glacihenhaus N, Gesnel M and Breathnach R (1985) Epidermal growth factor and oncogenes induce transcription of the same cellular mRNA in rat fibroblasts. *EMBO J* **4**: 1435–1440

McDonnell S, Navre M, Coffey RJ Jr and Matrisian LM (1991) Expression and localization of the matrix metalloproteinase PUMP-1 (MMP-7) in human gastric and colon carcinomas. *Mol Carcinogen* **4**: 527–533

Meade-Tollin L, Bowen CP, Fusenig N and Bowden GT (1992) Matrix metalloproteinase mRNA expression in C-Ha-ras transfected human keratinocytes (Abstract). *Proc Am Assoc Cancer Res* **33**: 68

Muller D, Quantin B, Gesnel MC, Millon-Collard R, Abecassis J and Breathnach R (1988) The collagenase gene family in humans consists of at least four members. *Biochem J* **253**: 187–192

Muller DB, Breathnach R, Engelmann A, Millon R, Bronner G, Flesch H and Dumont P, Eber M, Abecassis J (1991) Expression of collagenase-related metalloproteinase genes in human lung or head and neck tumors. *Int J Cancer* **481**: 550–556

Pajouh MS, Nagle RB, Breathnach R, Finch JS, Brawer MK and Bowden GT (1991) Expression of metalloproteinase genes in human prostate cancer. *J Cancer Res Clin Oncol* **117**: 1–7

Peltz SW, Brewer G, Bernstein P, Hart PA and Ross J (1991) Regulation of mRNA turnover in eukaryotic cells. *Crit Rev Eukaryotic Gene Expression* **1**: 99–126

Pendás AM, Knäuper V, Puente XS, Llano E, Mattei M, Apte S, Murphy G and Lopez-Otin C (1997) Identification of characterization of a novel human matrix metalloproteinase with unique structural characteristics, chromosomal location, and tissue distribution. *J Biol Chem* **272**: 4281–4286

Petersen MJ, Woodley D, Stricklin G and O'Keefe E (1989) Constitutive production of procollagenase and tissue inhibitor of metalloproteinases by human keratinocytes in culture. *J Invest Dermatol* **92**: 156–159

Polette M, Clavel C, Muller D, Abecassis J, Binninger I and Birembaut P (1991) Detection of mRNAs encoding collagenase I and stromelysin 2 in carcinomas of the head and neck by in situ hybridization. *Invasion Metastasis* **11**: 76–83

Powell WC, Knox JD, Navre M, Grogan TM, Kittelson J, Nagle RB and Bowden GT (1993) Expression of the metalloproteinase matrilysin in DU-145 cells increases their invasive potential in severe combined immunodeficient mice. *Cancer Res* **53**: 417–422

Pyke C, Ralfkiaer EH, Huhtala P, Hurskainen T, Dano K and Tryggvason K (1992) Localization of messenger RNA for Mr 72,000 and 92,000 type IV

© Cancer Research Campaign 1998 

collagenases in human skin cancers by *in situ* hybridization. *Cancer Res* **52**: 1336–1341

Quantin B, Murphy G and Breathnach R (1989) Pump-1 cDNA codes for a protein with characteristics similar to those of classical collagenase family members. *Biochemistry* **28**: 5327–5334

Ray JM and Stetler-Stevenson WG (1994) The role of matrix metalloproteases and their inhibitors in tumor invasion, metastasis, and angiogenesis. *Eur Resp J* **7**: 2062–2072

Rhim JS, Yoo JH, Park JH, Thraves P, Salehi Z and Dritschillo A (1990) Evidence for the multi-step nature of *in vitro* human epithelial cell carcinogenesis. *Cancer Res* **50**: 5653s–5657s

Roop DR, Lowy DR, Tambourin PE, Strickland J, Harper JR, Balaschak M, Spangler EF and Yuspa SH (1986) An activated harvey ras oncogene produces benign tumors on mouse epidermal tissue. *Nature* **323**: 822–824

Sarret Y, Woodley D, Goldberg G, Kronberger A and Wynn KC (1992) Constitutive synthesis of a 92-kDa Type IV collagenase is enhanced by Type I collagen and decreased by Type IV collagen matrices. *J Invest Dermatol* **99**: 836–841

Shapiro S, Doyle G, Ley T, Parks W and Welgus H (1993) Molecular mechanisms regulating the production of collagenase and TIMP in U937 cells: Evidence for involvement of delayed transcriptional activation and enhanced mRNA stability. *Biochemistry* **32**: 4286–4292

Shima I, Sasaguri Y, Kusukawa J, Nakano R, Yamana H, Fujita H, Kakegawa T and Morimatsu M (1993) Production of matrix metalloproteinase 9 (92-kDa gelatinase) by human oesophageal squamous cell carcinoma in response to epidermal growth factor. *Br J Cancer* **67**: 721–727

Smith, JA (1994) Quantitation of proteins. Ausubel FB, Brent R, Kingston RL, Moore DD, Smith JA, Seidman JG and Struhl K (eds), 10.1.1–10.1.3. Greene Publishing Associates and Wiley-Interscience. New York.

Stetler-Stevenson W (1990) Type IV collagenases in tumor invasion and metastasis. *Cancer Metast Rev* **9**: 289–303

Thraves P, Salehi Z, Dritschillo A and Rhim JS (1990) Neoplastic transformation of immortalized human epidermal keratinocytes by ionizing radiation. *Proc Natl Acad Sci USA* **87**: 1174–1177

Tryggvason K, Hoyhtya M and Pyke C (1993) Type IV collagenases in invasive tumors. *Breast Cancer Res Treat* **24**: 209–218

Wilhelm SM, Collier IE, Marmer BL, Eisen AZ, Grant GA and Goldberg GI (1989) SV40-transformed human lung fibroblasts secrete a 92-kDa type IV collagenase which is identical to that secreted by normal human macrophages. *J Biol Chem* **264**: 17213–17221

© Cancer Research Campaign 1998